# Role of DNA methylation in regulating inflammatory cytokine expression in neonates with late-onset sepsis

**DOI:** 10.3389/fimmu.2025.1613333

**Published:** 2026-01-26

**Authors:** Saranya Sankar, Kathirvel Maruthai, Zachariah Bobby, Bethou Adhisivam

**Affiliations:** 1 Department of Neonatology, Jawaharlal Institute of Postgraduate Medical Education and Research (JIPMER), Puducherry, India; 2 Department of Paediatrics, Jawaharlal Institute of Postgraduate Medical Education and Research (JIPMER), Puducherry, India; 3 School of Medicine, Department of Infectious Diseases, Johns Hopkins University, Baltimore, MD, United States; 4 Department of Biochemistry, Jawaharlal Institute of Postgraduate Medical Education and Research (JIPMER), Puducherry, India

**Keywords:** neonatal sepsis, DNA methylation, inflammatory cytokines, infection, gene expression regulation

## Abstract

**Background:**

Neonatal sepsis is a major health issue, particularly in resource-poor areas. This study aimed to assess the role of DNA methylation in regulating the inflammatory cytokine expression in late-onset neonatal sepsis (LOS).

**Methodology:**

We conducted a cross-sectional study comparing neonates with LOS (n=42) before and after 72 hours of antibiotic treatment (n=25) to healthy controls (HC) (n=42). We analysed DNA methylation patterns and inflammatory cytokine expression levels in both groups.

**Results:**

DNA methylation analysis revealed hypomethylation in pro-inflammatory genes and hypermethylation in anti-inflammatory genes in LOS neonates. These patterns were shifted in follow-up group, with significant changes in inflammatory marker levels, including TNF-α, IFN-γ, IL-10, and TGF-β. The gene expression results aligned with the DNA methylation findings, indicating that changes in the expression of inflammatory genes in late-onset neonatal sepsis (LONS) are influenced by DNA methylation. The results were further validated through an analysis of plasma cytokine levels. LOS cases showed elevated pro-inflammatory cytokines and reduced anti-inflammatory cytokines, except IL-10. In the follow-up group, pro-inflammatory cytokine levels decreased, while anti-inflammatory cytokines increased.

**Conclusion:**

DNA methylation regulates the expression of inflammatory cytokine genes in late-onset neonatal sepsis. Understanding these molecular changes could lead to better diagnostic and therapeutic approaches for managing neonatal sepsis.

## Introduction

Neonatal sepsis involves a compromised host immune response to microbes and their byproducts, leading to biochemical irregularities and organ dysfunction (1). Timely diagnosis poses a significant challenge in resource-constrained settings (2). Organ dysfunction arises from an imbalanced immune reaction to the infection within the host (2, 3). In sepsis, infections occurring within the first 72 hours are termed early-onset sepsis (EOS), while those manifesting after 72 hours of birth are referred to as late-onset sepsis (LOS) (1–4).

In this context, innate immune cells, such as neutrophils and monocytes, display heightened immune responses to invading pathogens, characterized by increased secretion of pro-inflammatory cytokines (5–8). The intensity of the initial inflammatory response in sepsis is influenced by a combination of host genetic factors, pathogen load, and virulence. Because of this pro-inflammatory reaction, there is a subsequent release of anti-inflammatory cytokines, contributing to a state of immunosuppression (7–11).

The initial line of defense against bacterial infections is orchestrated by pattern recognition receptors (PRRs) within innate immune cells (10). When bacterial antigens bind to PRRs, this triggers the activation of immune cells, resulting in significant alterations in the transcriptional activation of immune response genes, including various inflammatory cytokines and chemo attractants. These changes aim to provide a robust protective immune response within the host (10, 12–14). The equilibrium between pro-inflammatory and anti-inflammatory cytokines, as well as the involvement of specific immune cells, plays a crucial role in determining the host’s ability to effectively clear pathogens (15, 16). Epigenetic mechanisms influence the expression of inflammatory cytokine genes by modifying them without changing the DNA sequence, potentially altering the host’s immune response during infection (17). This may lead to immunosuppression in neonatal sepsis.

The impact of DNA methylation on the expression of immune response genes in neonatal sepsis remains unexplored, necessitating further investigation. Therefore, this study was undertaken to unveil and examine the influence of DNA methylation changes on the expression of inflammatory immune genes in response to late-onset neonatal sepsis.

## Methodology

### Patient details

This cross-sectional comparative study was conducted following approval from the Institute Ethics Committee (ethical review no. JIP/IEC2019/0125). Informed consent was obtained from the parents of the enrolled neonates. The sample size was determined using OpenEpi to achieve a reliable difference in DNA methylation levels between the two groups with 5% significance and 95% power, factoring in a 25% dropout rate (18). As a result, a total of forty-two (n=42) neonates with late-onset sepsis (LOS) and forty-two (n=42) healthy neonates were included in the study.

The study included neonatal sepsis based on the presence of two or more clinical signs and symptoms, including difficulty feeding or feeding intolerance, abdominal distension, convulsions, abnormal posturing, hypotonia, no or minimal movement on stimulation, lethargy or drowsiness, irritability, bulging fontanelle, pus from the umbilical stump, abnormal temperature (>37.5 °C or <36.5 °C) or temperature instability, abnormal heart rate (>180/min or <100/min), severe chest in-drawing, increased oxygen requirement or need for ventilation support, grunting, apnea, capillary refill time (CRT) > 3 seconds, mottled skin or other evidence of shock, and cyanosis. Laboratory markers used for diagnosis included white blood cell (WBC) count < 4 × 10^9^ cells/L or > 20 × 10^9^ cells/L, absolute neutrophil count < 1.5 × 10^9^ cells/L, and C-reactive protein (CRP) > 10 mg/L or > 1 mg/dL. Since the blood samples were collected based on sepsis screening, the 42 neonates were classified as cases based on positive blood cultures, while those with negative cultures were classified as having clinical sepsis (n=23). Follow-up was conducted for 25 of the 42 cases due to study dropouts. The exclusion criteria for both cases and controls encompassed major congenital malformations, neonatal demise within 6 hours of admission, neonates weighing less than 1000 grams, and those with a gestational age of less than 30 weeks. Blood samples from the cases were collected both before initiating antibiotic treatment and after 72 hours of antibiotic treatment. The aim of the study was to analyze the inflammatory profile before starting antibiotic treatment and after 72 hours of antibiotic treatment. Since the sample collected before starting of antibiotics with suspicion of sepsis, the neonates with positive blood culture were considered as cases and the neonates with negative blood culture with signs and symptoms of sepsis were considered as clinical sepsis. Peripheral blood samples (2 mL each) were collected at the onset of suspected sepsis and again 72 hours after initiation of antibiotic treatment from newborns, with parental informed consent obtained during routine laboratory blood sampling. For DNA isolation whole blood sample of 0.5ml and RNA isolation 0.5ml was used. Remaining 1.0ml of blood sample was used for plasma separation at 3000 rpm for 10 minutes. After sample collection, the samples undergo separation into plasma, genomic DNA, and serum, which are then stored at -80 degrees Celsius. Subsequently, a batch analysis is conducted to eliminate any processing errors, and the results are generated once all the samples have been processed accordingly.

Healthy neonates, matched in terms of gestational age and gender, and undergoing blood collection for conditions such as thyroid screening, were selected as controls.

### DNA methylation analysis

#### Bisulfite conversion

The bisulfite conversion process was conducted following the guidelines of the EZ DNA Methylation Gold Kit (Zymo Research Inc., USA). Briefly, 500 ng of genomic DNA was subsequently extracted from the whole blood using a FavorGen genomic DNA isolation kit (19) from cases and controls underwent bisulfite conversion, which distinguishes unmethylated Cs by converting them to Us, while leaving methylated Cs unaffected. The converted DNA was subsequently stored at -20 °C.

#### CpG Island location and primer design

The consensus sequence was obtained from PubMed and a publicly accessible source at TranspoGene (https://www.ensembl.org/index.html). To identify CpG islands (CGI) in the promoter region, the DBCAT tool (http://dbcat.cgm.ntu.edu.tw/) was utilized. Methylation-specific polymerase chain reaction (MS-PCR) primers were designed using the MethPrimer tool with default settings. Additional information regarding the primers is available in **Supplementary Table S1**.

#### Methylation-specific polymerase chain reaction

For the MS-PCR analysis, separate reactions were carried out to assess both M (methylated) and U (unmethylated) components. In each case, a 20 μL PCR mixture was prepared, comprising 1 μL of bisulfite-converted DNA, 1 μL of each forward and reverse primer (100 pmoL) specific to either the methylated or unmethylated sequences (separate for each reaction), 8 μL of ZymoTaq master mix, and 9 μL of nuclease-free water. The PCR cycle consisted of an initial denaturation at 95°C for 10 minutes, followed by 35 cycles, each involving denaturation at 95°C for 30 seconds, annealing at 57°C for 35 seconds, extension at 72°C for 30 seconds, and a final extension at 72°C for 10 minutes.

### Methylation standard DNA preparation

Commercially purchased fully methylated DNA (100% M) and fully unmethylated DNA (100% U) (Qiagen, Germany) were used as positive and negative controls respectively. The standard concentrations of DNA methylation were prepared as follows 0%, 5%, 10%, 25%, 50%, 75%, and 100% by mixing both M and U DNA to prepare a standard curve.

#### Agarose gel electrophoresis and quantification

We performed a 1.5% agarose gel electrophoresis to separate the PCR-amplified products obtained using both methylated and unmethylated primers. The standards included samples with 0%, 10%, 25%, 50%, 75%, and 100% methylation levels. The band intensities, representing the presence or absence of M and U MS-PCR products, were visualized using the ImageQuant LAS 500 system (GE Healthcare, UK) and subsequently compared with the control standards. [19].

### Gene expression analysis

#### RNA extraction, and cDNA synthesis

The total RNA was extracted from whole blood using the TRIzol manual method (Chomczynski and Sacchi 1987). In a 2 mL centrifuge tube, an equal volume of whole blood (500 μL) and TRIzol reagent (500 μL) were combined for total RNA isolation. The resulting RNA pellet was dissolved in 50 μL of Nuclease-free sterile water and stored at -20 °C until further use. The purity and concentration of the RNA were evaluated using a NanoDrop 2000 spectrophotometer (ThermoFisher Scientific, MA, USA). To eliminate any residual genomic DNA from the total RNA, RNase-free DNase I (ThermoFisher Scientific, MA, USA) was used following the manufacturer’s instructions.

One microgram (1µg) of total RNA was used for complementary DNA (cDNA) synthesis by using the RevertAid H Minus First Strand cDNA Synthesis kit (ThermoFisher Scientific, USA) according to the manufacturer’s recommendation.

#### qRT-PCR analysis

We procured gene-specific primers for TLR2, TLR4, IL-1β, TNF-α, IFN-γ, IL-6, CXCL-1, IL-10, TGF-β, and FOXP3 from IDT (USA). The total volume for the qRT-PCR reaction consisted of 2 μL of cDNA (diluted at a ratio of 1:30), 10 μL of Takara SYBR master mix (TB Green Premix Ex Taq II Tli RNase H Plus, Takara Bio Inc., Japan), 0.4 μL of gene-specific primers at a concentration of 200 nM, and 7.2 μL of RNase-free water (Clontech Laboratories, Inc, CA, USA). The qRT-PCR was carried out using the CFX96 Real-Time PCR Detection System (Bio-Rad, USA). The following reaction conditions were applied: initial denaturation at 95 °C for 30 seconds, followed by 40 cycles of denaturation at 95 °C for 5 seconds and annealing/extension at 60 °C for 30 seconds. To verify target-specific amplification, a melt-curve analysis was conducted in the range of 60 °C to 90 °C at a rate of 0.2 °C per minute, with fluorescence intensity observed through the CFX96 Real-Time PCR Detection System (Bio-Rad, USA). Each reaction was performed in duplicates at least, and the human acidic ribosomal protein (HuPO) gene served as a housekeeping gene for normalizing the cycle threshold (Ct) values in both the test and control samples. To assess the expression levels of inflammatory genes in both the case and control groups, we employed a comparative Ct method (2-delta Ct). Additional details regarding the primers are provided in **Supplementary Table S2**.

### Quantification of pro- and anti-inflammatory cytokines in neonates

The EliKine™ (Abbkine) Human IFN-γ, TNF-α, IL-10, and TGF-β ELISA kit employs a sandwich ELISA approach to quantify cytokine levels in samples. In this method, plates are pre-coated with specific antibodies for each cytokine. Standards and samples are subsequently pipetted into the wells, allowing the cytokines (IFN-γ range 7.8 pg/mL-500 pg/mL, TNF-α, range 7.8 pg/ml-500 pg/ml, IL-10 range 2.35 pg/ml-150 pg/ml, and TGF-β range 15.6 pg/ml-1000 pg/ml) present in the sample to bind to the immobilized antibodies. Following the removal of any unbound substances, a biotin-conjugated antibody, specific to these cytokines (IFN-γ, TNF-α, IL-10, and TGF-β), is introduced to the wells. Proprietary EliKine™ Streptavidin-HRP conjugates are then added to the wells to bind with unbound streptavidin-enzyme reagents. Finally, a substrate solution is added to the wells, and the color developed is directly proportional to the amount of these cytokines initially bound. The intensity of the color is subsequently measured for quantification.

### Statistical analysis

Shapiro-Wilk test was used to assess the normality distribution. Categorical data were expressed as numbers and percentages. Results of all parameters were presented as median with Interquartile range (IQR). Mann-Whitney U test was used to compare the expression levels of inflammatory genes, and DNA methylation level between the groups. Wilcoxon matched-pairs signed-rank test was used to compare between the follow-up groups. All statistical analyzes were carried out in SPSS 17 (SPSS, Chicago, IL), GraphPad Prism 6.0 (GraphPad Software Inc., CA), and MS-Excel at a 95% confidence interval with *p* < 0.05 was considered as significant.

## Results

The study included 42 neonates with culture-proven late-onset sepsis (LOS), along with an equal number of healthy controls. Follow-up assessments were conducted in 25 of the 42 cases. Both groups exhibited similar baseline characteristics, as shown in **Table 1**. Based on the blood culture report, most of the organisms are identified as gram-negative bacteria. Blood culture analysis showed *K. pneumonia* in 31%, *Elizabethkingia* sp. in 29%, *A. baumannii* in 14%, *Enterobacter* sp. in 9.5%, *Citrobacter* sp. in 4.7%, *E. coli* in 2.4%, *Methicillin-Resistant Staphylococcus aureus* in 4.7%, and *P. aeruginosa* in 4.7%. Of them, 14% cases showed combined bacterial infections like *Enterobacter* sp*, Citrobacter* sp*, A. baumannii*, and *P. aeruginosa* and 12 (28.5%) cases had a recurrent infection. Increased *Elizabethkingia* sp. isolated in our study was due to a nosocomial outbreak during the study period. The hematological investigation of 42 neonates with culture-proven late-onset sepsis (LOS), showed Leukopenia in 10 cases and Leukocytosis in 18 cases.

**Table 1:** Baseline characteristics of cases and controls.

S. No	Characteristics	Cases n = 42 Frequency (%)	Controls n = 42 Frequency (%)
1.	Gender		
	Male	24 (57%)	23 (55%)
	Female	18 (43%)	19 (45%)
2.	Mode of Delivery		
	SVD	31 (73%)	35 (83%)
	LSCS	11 (27%)	7 (17%)
3.	Gestational Age		
	Term (>37 weeks)	16 (38%)	22 (53%)
	Preterm (30–36 weeks)	26 (62%)	20 (47%)
4.	Birth Weight		
	Low birth weight 1500 - 2500 (gms)	24 (57%)	20 (47%)
	Normal birth weight >2500 (gms)	18 (43%)	22 (53%)
5.	Liquor		
	Clear	30 (72%)	32 (76%)
	MSL	12 (28%)	10 (24%)

The baseline characteristics are represented in frequency (%); n – Numbers; % - Percentage; gms – Grams; SVD – Spontaneous vaginal delivery, LSCS - Lower segment caesarean section; MSL - Meconium-stained liquor; LBW – Low birth weight; NBW – Normal birth weight.

### Gene-specific DNA methylation patterns of pro- and anti-inflammatory genes

**Table 2** presents the comparison of DNA methylation levels for pro- and anti-inflammatory genes among culture-positive sepsis, clinical sepsis, and controls, with results expressed as Median with IQR. The selected candidate genes displayed a significant difference in DNA methylation levels between the groups (p<0.0001). Notably, TLR-2 (p=0.9), TGF-β (p=0.1), and FOXP3 (p=0.2) did not exhibit significant differences between culture-positive and clinical sepsis. However, TGF-β and FOXP3 exhibited a hypermethylation pattern in culture-positive and clinical sepsis compared to controls (p<0.0001). A significant difference was observed in DNA methylation levels between clinical sepsis and controls, as well as between the three groups. Overall, there was a trend of hypomethylation in pro-inflammatory genes and hypermethylation in anti-inflammatory genes, except for the IL-10 gene, which displayed hypomethylation in culture-positive and clinical sepsis compared to the control group. **Supplementary Figure S1** provides a representative agarose gel image illustrating the percentage of DNA methylation in pro- and anti-inflammatory cytokine genes among the neonates with late-onset neonatal sepsis (LONS) and healthy controls enrolled in this study.

**Table 2:** mRNA levels of pro-inflammatory cytokines from culture-positive, clinical sepsis, and healthy controls.

Gene	Culture + sepsis (A)	Clinical sepsis (B)	*p* value (A vs B)	Controls (C)	*p* value (A vs C)	*p* value (B vs C)	*p* value (Kruskal-Wallis)
*TLR-2*			0.9		<0.0001	<0.0001	<0.0001
Median	25	25		75			
IQR	10-25	10-25		50-75			
*TLR-4*			<0.0001		<0.0001	<0.0001	<0.0001
Median	10	25		62.5			
IQR	10-25	25-50		50-75			
*TNF-α*			<0.0001		<0.0001	<0.0001	<0.0001
Median	10	50		50			
IQR	10-25	25-50		50-75			
*IFN-γ*			<0.0001		<0.0001	<0.0001	<0.0001
Median	10	25		50			
IQR	10-25	25-50		50-75			
*IL-1β*			<0.0001		<0.0001	<0.0001	<0.0001
Median	10	25		50			
IQR	10-25	25-50		50-75			
*IL-6*			0.004		<0.0001	<0.0001	<0.0001
Median	10	25		75			
IQR	10-25	10-25		50-75			
*CXCL1*			<0.0001		<0.0001	<0.0001	<0.0001
Median	10	25		50			
IQR	10-25	25-50		50-75			
*IL-10*			<0.0001		<0.0001	0.001	<0.0001
Median	25	50		75			
IQR	10-25	50-75		50-75			
*TGF-β*			0.1		<0.0001	<0.0001	<0.0001
Median	75	75		25			
IQR	50-75	75-75		25-25			
*FOXP3*			0.2		0.02	0.004	0.007
Median	75	75		50			
IQR	50-75	75-75		50-75			

Mann Whitney U test was used to compare the gene expression levels between culture + vs clinical sepsis. Kruskal-Wallis test was used to compare the gene expression levels between culture + vs clinical sepsis vs controls. Data are mentioned in median with interquartile range. *p*-value, < 0.05.

**Supplementary Tables S3A, B** detail the comparison of DNA methylation levels for pro- and anti-inflammatory genes within subgroups, such as gestational age, birth weight, and disease outcome. IL-6 (p=0.04) displayed a significant difference between survivors and non-survivors. Additionally, TGF-β (p=0.02) exhibited a significant difference between pre-term and term groups, with hypermethylation in the pre-term group compared to the term group. However, other genes did not exhibit significant differences in any of the subgroups.

In the follow-up group, aside from TLR-4 (p=0.08), CXCL1 (p=0.9), and IL-10 (p=0.7), all other genes showed significant differences. Specifically, hypermethylation in pro-inflammatory genes and hypomethylation in anti-inflammatory genes were observed in pre-treatment group compared to post-treatment group (**Table 3**). Notably, the differences were less pronounced in TLR-4 (p=0.002), TNF-α (p=0.01), and IFN-γ (p=0.009) compared to TLR-2, IL-1β, TGF-β, and FOXP3 (p<0.0001), which exhibited greater disparities between the groups.

**Table 3:** Comparison of inflammatory gene expression in follow-up cases.

Group	No	% Methylation (median, IQR)									
		*TLR2*	*TLR4*	*TNF-α*	*IFN-γ*	*IL-1β*	*IL-6*	*CXCL1*	*IL-10*	*TGF-β*	*FOXP3*
Pre treatment	25	10 (10-25)	10 (10-21.25)	25 (10-25)	25 (10-25)	10 (10-25)	10 (10-25)	10 (10-25)	25 (10-25)	75 (50-75)	75 (50-75)
Post treatment	25	50 (25-50)	17.5 (10-25)	25 (25-50)	25 (25-50)	25 (25-50)	25 (10-50)	10 (10-25)	10 (10-25)	25 (25-50)	25 (10-25)
*P* value		<0.0001	0.08	0.01	0.009	<0.0001	0.002	0.9	0.7	<0.0001	<0.0001

Mann Whitney U test was used to compare the gene expression levels of inflammatory genes between pre-treatment and post treatment group. Data are mentioned in median with interquartile range. *p*-value, < 0.05.

### Expression profile of candidate pro- and anti-inflammatory genes

In compliance with the inclusion and exclusion criteria, we included a total of forty-two neonates with late-onset sepsis (57% males, 43% females) and forty-two healthy control (HC) neonates, matched for gestational age and gender (55% males, 45% females). We also included twenty-three neonates with clinical sepsis, despite negative blood cultures. **Table 4** offers a comparison of pro- and anti-inflammatory gene expression among culture-positive sepsis (n=42), clinical sepsis (n=23), and controls (n=42). All genes demonstrated significant differences between the groups (p<0.0001), except for TLR-2 (p=0.2), IFN-γ (p=0.2), TGF-β (p=0.4), and FOXP3 (p=0.1), which did not exhibit a significant difference between culture-positive and clinical sepsis. Notably, TGF-β, and FOXP3 expression was lower in culture-positive sepsis compared to clinical sepsis. TGF-β and FOXP3 displayed lower expression in both culture-positive and clinical sepsis compared to controls (p<0.0001). Overall, higher expression of pro-inflammatory genes and lower expression of anti-inflammatory genes were observed in culture-positive and clinical sepsis compared to controls, except for IL-10, which exhibited higher expression than controls.

**Table 4:** Comparison of % DNA methylation levels of pro-inflammatory genes in late-onset neonatal sepsis cases and controls.

Gene	Culture + sepsis (A)	Clinical sepsis (B)	*p* value (A vs B)	Controls (C)	*p* value (A vs C)	*p* value (B vs C)	*p* value (Kruskal-Wallis)
*TLR-2*			0.2		<0.0001	0.0006	<0.0001
Median	4.4	3.87		2.7			
IQR	3.4-5.7	2.8-6.1		1.9-3.6			
*TLR-4*			<0.0001		<0.0001	<0.0001	<0.0001
Median	6.8	3.5		1.9			
IQR	4.3-10.6	2.8-4.5		1.5-2.9			
*TNF-α*			0.004		<0.0001	<0.0001	<0.0001
Median	5.47	4.4		1.95			
IQR	4.4-7.5	3.0-5.6		1.7-3.1			
*IFN-γ*			0.2		<0.0001	0.0008	<0.0001
Median	6.73	6.09		4.42			
IQR	4.9-9.3	3.8-8.4		3.4-5.0			
*IL-1β*			0.01		<0.0001	0.003	<0.0001
Median	5.71	3.8		1.98			
IQR	4.2-7.8	1.7-6.9		1.3-2.7			
*IL-6*			0.001		<0.0001	<0.0001	<0.0001
Median	8.14	5.6		3.05			
IQR	5.7-11.8	3.5-8.2		2.4-4.2			
*CXCL1*			<0.0001		<0.0001	0.0002	<0.0001
Median	5.41	2.7		1.68			
IQR	3.8-6.9	1.9-3.4		1.3-2.3			
*IL-10*			0.02		<0.0001	<0.0001	<0.0001
Median	4	2.9		1.9			
IQR	2.7-5.4	2.5-3.6		1.5-2.5			
*TGF-β*			0.4		<0.0001	<0.0001	<0.0001
Median	1.9	2.2		4.2			
IQR	1.4-2.6	1.3-2.6		3.7-4.6			
*FOXP3*			0.1		<0.0001	0.03	0.0005
Median	1.8	2.6		2.9			
IQR	1.3-2.8	1.5-2.9		2.1-3.8			

Mann Whitney U test was used to compare the % DNA methylation level between culture + vs clinical sepsis. Kruskal-Wallis test was used to compare the % DNA methylation level between culture + vs clinical sepsis vs controls. Data are mentioned in median with interquartile range. *p*-value, < 0.05.

Further, an analysis of the expression levels of pro- and anti-inflammatory cytokines within sub-groups based on gestational age, birth weight, and disease outcome (**Supplementary Tables S4A, B**) were performed. IFN-γ (p=0.05) and IL-6 (p=0.01) showed a significant difference between survivors and non-survivors, with higher expression levels in the non-survivor group. A significantly lower expression of TGF-β (p=0.03) was observed in the preterm group compared to the term group, while other genes did not display significant expression differences.

**Table 5** present the expression of pro- and anti-inflammatory genes in the follow-up group of culture-positive sepsis, comparing pre-treatment (under 12 hours) and post-treatment (72 hours) with standard antibiotic therapy. TLR-4 (p=0.2), and CXCL1 (p=0.6) did not show significant differences during the follow-up, while other genes exhibited significant differences. Pro-inflammatory genes displayed lower expression in the post-treatment group compared to the pre-treatment group, whereas anti-inflammatory gene expression was higher in the post-treatment group. Notably, IL-10 expression was further elevated in the post-treatment group.

**Table 5:** Comparison of % DNA methylation of inflammatory genes in follow-up cases.

Group	No	Gene expression, median (IQR)										
		*TLR2*	*TLR4*	*NFκB*	*TNF-α*	*IFN-γ*	*IL-1β*	*IL-6*	*CXCL1*	*IL-10*	*TGF-β*	*FOXP3*
Pre treatment	25	4.6 (4.2-6.0)	7.1 (4.9-10.7)	5.1 (3.2-8.1)	5.1 (3.9-6.9)	7.9 (5.3-9.3)	6.5 (4.1-8.8)	7.4 (4.6-10.6)	5.0 (3.3-6.5)	3.8 (2.7-6.3)	1.6 (1.3-2.2)	1.8 (1.0-3.0)
Post treatment	25	3.7 (2.6-4.2)	6.5 (5.0-8.0)	5.1 (4.2-6.6)	3.7 (2.5-5.0)	5.4 (4.1-6.9)	3.5 (2.5-6.2)	4.6 (2.5-6.5)	4.8 (4.2-6.2)	5.4 (4.2-6.6)	7.7 (5.9-7.7)	4.19 (3.3-5.6)
*p* value		0.0005	0.2	0.7	0.005	0.007	0.003	0.003	0.6	0.04	<0.0001	<0.0001

Mann Whitney U test was used to compare the % DNA methylation levels of inflammatory genes between pre-treatment and post treatment group. Data are mentioned in median with interquartile range. *p*-value, < 0.05.

### Plasma levels of pro- and anti-inflammatory cytokines in neonates with LONS and controls

**Figure 1:**
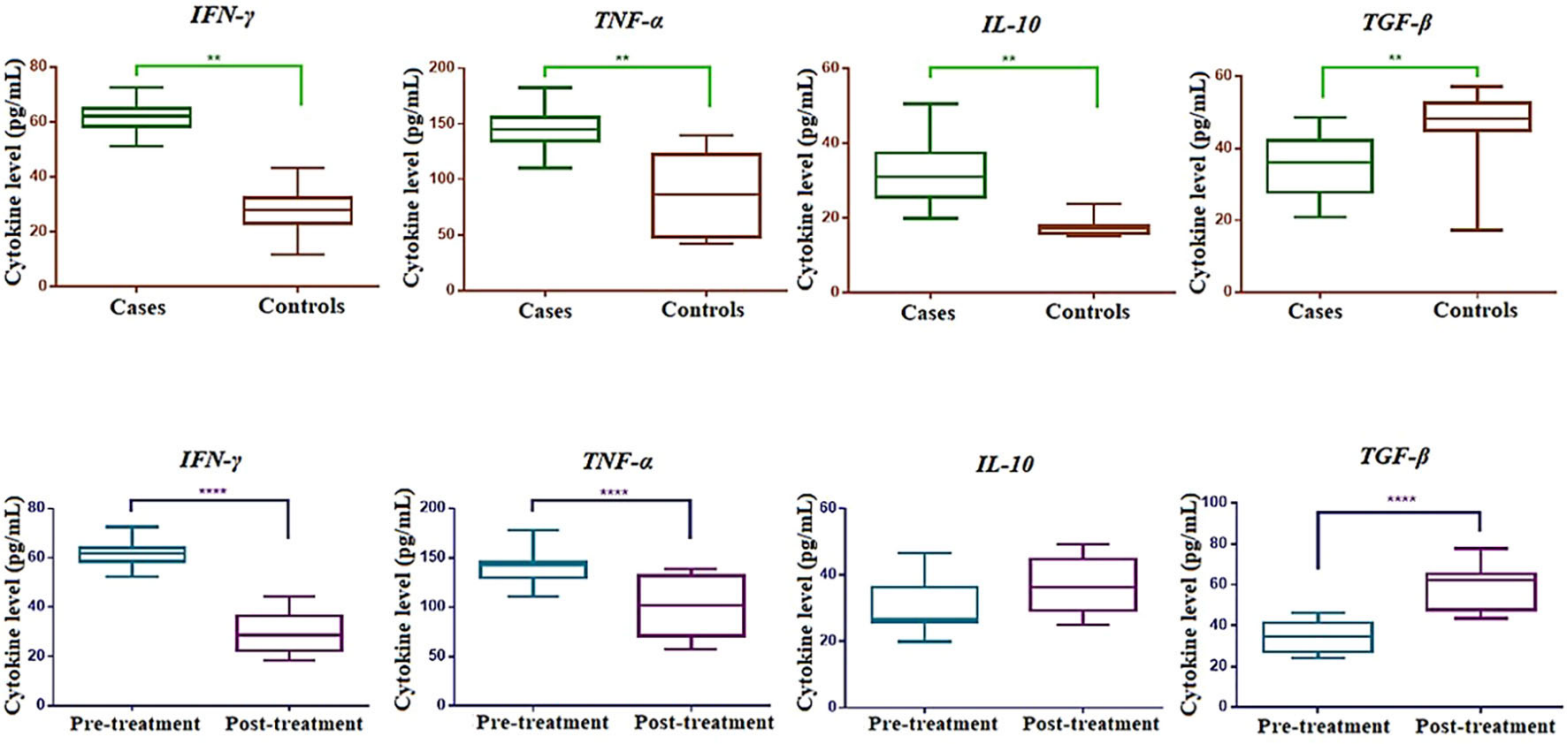
Cytokine levels in plasma of neonates with late-onset neonatal sepsis (LONS) and controls, including follow-up data.

**Figure 2:**
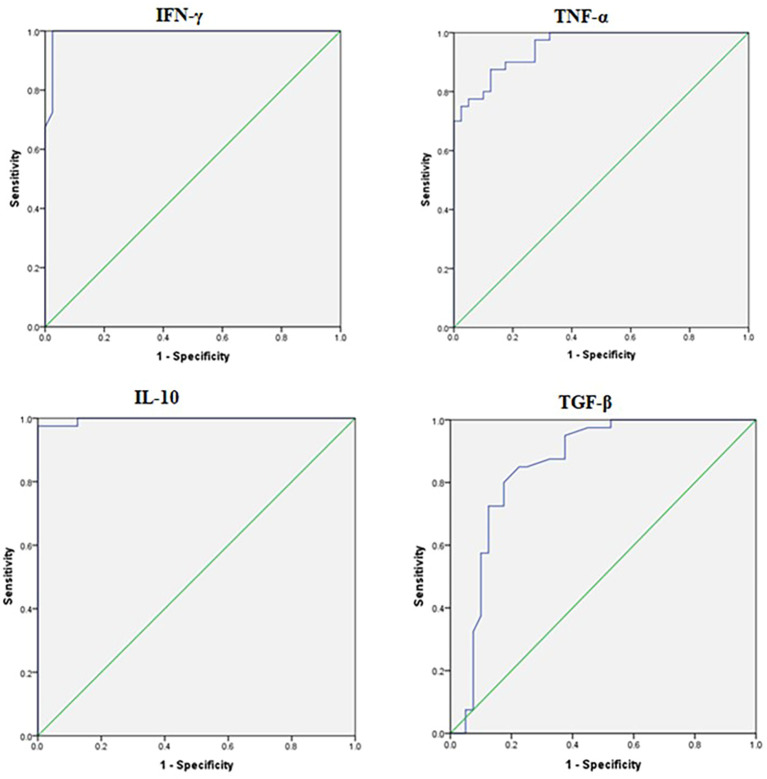
ROC analysis of inflammatory cytokines. Diagnostic performance of plasma cytokines IFN-γ, TNF-α, IL-10 and TGF-β in differentiating cases and controls group; AUC, Area under the curve; *p*-value, <0.05.

**Figure 1** illustrates the plasma levels of selected inflammatory cytokines, including TNF-α, IFN-γ, IL-10, and TGF-β, in culture-positive sepsis (n=42) and healthy controls (n=42). A significant difference was observed between the two groups, with higher levels of TNF-α, IFN-γ, and IL-10, and lower levels of TGF-β in culture-positive sepsis compared to controls. These findings align with the gene expression and methylation results in our study. In the follow-up, the analysis between pre and post treatment (**Figure 1**), TNF-α and IFN-γ levels were reduced, and TGF-β levels increased in culture-positive sepsis compared to controls. However, no significant difference was observed in IL-10 levels. ROC analysis demonstrated that TNF-α, IFN-γ, IL-10, and TGF-β could serve as effective diagnostic criteria for distinguishing culture-positive sepsis from healthy controls (**Supplementary Table S5**). **Figure 2** depicts the ROC analysis of pro- and anti-inflammatory cytokine levels. All cytokines, except for TGF-β, exhibited significantly higher levels in cases than in controls (p<0.0001).

## Discussion

This study provides comprehensive insights into the molecular and immunological landscape of late-onset sepsis (LOS) in neonates, emphasizing the interplay between gene-specific DNA methylation, gene expression, and cytokine profiles. The findings demonstrate a distinct pattern of immune dysregulation in culture-positive sepsis compared to healthy controls and clinical sepsis, reinforcing the role of epigenetic regulation in the pathophysiology of neonatal sepsis.

### Microbial profile and nosocomial influence

Blood culture analysis identified a predominance of gram-negative bacteria, with *Klebsiella pneumoniae* (31%) and *Elizabethkingia* sp. (29%) being the most common pathogens. The increased prevalence of *Elizabethkingia* sp. was attributed to a nosocomial outbreak during the study period (2). The diversity of pathogens, including *Acinetobacter baumannii*, *Enterobacter* sp., and *Pseudomonas aeruginosa*, along with combined infections in 14% of cases, underscores the complexity of microbial etiology in neonatal sepsis. The high recurrence rate (28.5%) highlights the challenge of eradicating pathogens in nosocomial settings and the need for stringent infection control measures.

### DNA methylation profiles and epigenetic modulation

DNA methylation analysis revealed significant differences in the methylation patterns of pro- and anti-inflammatory genes between culture-positive sepsis, clinical sepsis, and controls. A notable trend of hypomethylation in pro-inflammatory genes (e.g., TLR-2, TLR-4, IL-1β) and hypermethylation in anti-inflammatory genes (e.g., TGF-β, FOXP3) was observed in sepsis groups compared to controls. An experimental study by Gazzer et al. (2010) demonstrated that the TNF promoter was methylated and transcriptionally inactive in resting monocytes. However, during exposure to endotoxins, rapid demethylation occurred at the promoter region, exposing the transcription binding site for NFκB. As it progressed to endotoxin tolerance, rapid methylation of TNF promoters occurred due to the recruitment of DNMTs (20, 21). Interestingly, IL-10 exhibited hypomethylation in both culture-positive and clinical sepsis, suggesting a persistent pro-inflammatory state despite its immunomodulatory role. A study by Lorente-Pozo et al. (2021) showed hypomethylation of IL-10, IL-6, and IL-8 using a DNA methylation array in early and late-onset neonatal sepsis, supporting our findings (22). Lorente-Sorolla et al. (2019) demonstrated increased expression of IL-10 and IL-6 genes in adult septic patients with organ dysfunction and associated these changes with altered DNA methylation in monocytes (23). TLR-2, TGF-β, and FOXP3 did not exhibit significant differences between culture-positive and clinical sepsis, indicating shared epigenetic patterns between these groups. Subgroup analysis showed that IL-6 and TGF-β methylation levels varied significantly between survivors and non-survivors and between preterm and term neonates, respectively, highlighting the potential prognostic value of DNA methylation signatures.

### Gene expression patterns in culture-positive and clinical sepsis

The expression profiles of candidate pro- and anti-inflammatory genes further validated the methylation findings. Higher expression of pro-inflammatory genes (e.g., TLR-4, TNF-α, IL-1β, IFN-γ) and reduced expression of anti-inflammatory genes (e.g., TGF-β, FOXP3) were observed in both culture-positive and clinical sepsis groups compared to controls. Based on other studies, the infection may trigger the initiation of innate immune responses in neonates through the TLR-2 and TLR-4 signaling pathways, subsequently activating the transcription factor NFκB (15, 24, 25). Macrophages and monocytes play a pivotal role in releasing pro-inflammatory cytokines such as IL-1β, TNF-α, and IL-6 (26), which exhibit higher expression in cases of culture-positive sepsis. The elevated expression of IFN-γ in our study may play a role in activating macrophages and monocytes in response to infection during sepsis (27, 28). Furthermore, CXCL1 has the role in recruiting neutrophils to control infection and promotes the release of pro-inflammatory cytokines as cited by previous study (29–32). The increased expression of CXCL1 in our study indicates an increase in pro-inflammatory genes, consistent with the elevated leukocyte count observed in 64% of cases compared to controls. Notably, TGF-β and FOXP3 expression was lower in culture-positive sepsis cases than in clinical sepsis, suggesting a more pronounced suppression of regulatory pathways in culture-positive cases. IL-10, despite its anti-inflammatory role, exhibited higher expression in sepsis groups, likely reflecting a compensatory mechanism aimed at dampening excessive inflammation. Expression analysis in subgroups revealed significant differences in IFN-γ and IL-6 between survivors and non-survivors, with higher expression correlating with poor outcomes. Similarly, TGF-β expression was significantly lower in preterm neonates compared to term neonates, suggesting a compromised anti-inflammatory response in the preterm group. A recent study by Sherrianne et al. (2020) also revealed upregulation of TLR-2, TLR-4, IFN-γ, IL-6, IL-1β, and IL-10 expression in neonatal sepsis (33).

### Plasma cytokine profiles and diagnostic utility

Cytokine analysis corroborated the molecular findings, with significantly higher plasma levels of TNF-α, IFN-γ, and IL-10 and lower levels of TGF-β in culture-positive sepsis compared to controls. Post-treatment, TNF-α and IFN-γ levels decreased, and TGF-β levels increased, indicating a shift towards immune resolution. However, IL-10 levels did not show significant differences between pre- and post-treatment, reinforcing its sustained role in modulating immune responses during and after infection. T regulatory cells are involved in the secretion of IL-10 and TGF-β cytokines during inflammatory conditions. Previous studies have associated increased levels of TNF-α, IL-6, and IL-8 with severe sepsis and shock (34–38). In a neonatal mouse model of sepsis, the administration of IFN-γ at 18 hours resulted in improved survival and reduced bacterial counts in group B streptococcal infection, partially reversing the host’s defense against bacteria (39). Tissières et al. (2012) reported the reversal of immune response deficiency to bacteria in neonates with ex-vivo treatment of IFN-γ, which led to increased levels of IL-6 and TNF-α and decreased IL-10 levels (40). ROC analysis demonstrated that TNF-α, IFN-γ, IL-10, and TGF-β could serve as effective biomarkers for distinguishing culture-positive sepsis from healthy controls, with high sensitivity and specificity. ROC curves further highlighted the diagnostic value of these cytokines, with all markers except TGF-β exhibiting significantly higher levels in sepsis cases (p<0.0001).

### Impact of antibiotic treatment on methylation and gene expression

The follow-up analysis revealed significant differences in DNA methylation and gene expression levels pre- and post-treatment. Following antibiotic therapy, a shift towards hypermethylation in pro-inflammatory genes and hypomethylation in anti-inflammatory genes was observed, indicative of a resolution of the inflammatory response. The cytokine IL-6, extensively researched in neonatal sepsis for its short half-life, showed a significant decrease within 48 hours of treatment (41–44). Our study supports this finding, demonstrating a marked reduction in IL-6 expression in the post-treatment group. Notably, IL-10 expression remained elevated post-treatment, suggesting its sustained role in immune regulation during recovery. The increased expression of IL-10 in our study, secreted by various immune cells such as T cells, monocytes, macrophages, and NK cells (45), plays a complex role in neonatal sepsis. In adult sepsis, higher levels of IL-10 are associated with poor prognosis and septic shock. While IL-10 has a protective role in sepsis by suppressing pro-inflammatory cytokines, a high ratio of IL-10 to TNF-α has been linked to severe late-onset sepsis (45, 46). Our study indicated an increased expression of IL-10, along with lower expression of pro-inflammatory genes in the pre-treatment group (47–49). In the follow-up after 72 hours, there was an increase in IL-10, TGF-β, and FOXP3 expression, suggesting that immune suppression occurs in the later stages of sepsis.

The observed changes in gene expression align with the plasma cytokine levels, where TNF-α and IFN-γ decreased post-treatment, while TGF-β increased. These findings reinforce the dynamic nature of immune regulation during neonatal sepsis and recovery, emphasizing the potential role of epigenetic modifications as biomarkers for disease progression and treatment response.

### Clinical implications and future directions

The observed trends in DNA methylation, gene expression, and cytokine levels provide valuable insights into the immunopathogenesis of late onset neonatal sepsis. The interplay between pro- and anti-inflammatory responses, regulated through epigenetic modifications, highlights potential targets for early diagnosis and therapeutic intervention. The persistent elevation of IL-10 and the differential expression of key inflammatory mediators suggest that modulation of immune responses may be a promising strategy for improving outcomes in neonatal sepsis. Future research should explore the mechanistic underpinnings of these findings, particularly the role of DNA methylation in driving long-term immune dysregulation and its potential as a biomarker for sepsis prognosis and treatment response.

## Conclusion

This study underscores the role of DNA methylation in modulating inflammatory responses in neonates with LOS. The observed hypomethylation of pro-inflammatory genes and hypermethylation of anti-inflammatory genes suggest an epigenetic basis for the exaggerated inflammatory response in neonatal sepsis. The reversibility of these changes with treatment further highlights the potential of epigenetic modifications as both biomarkers and therapeutic targets in neonatal sepsis management. Further research is warranted to explore the clinical utility of these findings in improving neonatal sepsis outcomes.

## Data Availability

The data generated in this study consist of quantitative real-time PCR (qRT-PCR) and ELISA measurements. No large-scale sequencing, omics, or imaging datasets requiring deposition in public repositories were generated. All raw and processed qRT-PCR and ELISA data supporting the findings of this study are included within the article and/or its supplementary material and are available from the corresponding author upon reasonable request.

## Supplementary material

Supplementary Figure 1 Representative result of MS-PCR analysis –inflammatory Cytokine Genes. The Supplementary Figures S1 shows the presence of methylation band in agarose gel electrophoresis.

Supplementary Table 1 List of qRT-PCR Primers. The Supplementary Table S1 shows the primer details of pro- and anti-inflammatory genes and housekeeping genes.

Supplementary Table 2 List MS-PCR Primers. The Supplementary Table S2 shows the methylation primer details of pro- and anti-inflammatory genes and housekeeping genes.

Supplementary Table 3 List Bisulfite Pyrosequencing Primers. The Supplementary Table S2 shows the bisulfite pyrosequencing primer details of pro- and anti-inflammatory genes and housekeeping genes.

Supplementary Table 4 **(A)** Comparison of Pro-Inflammatory Gene Expression in Subgroups. Mann Whitney U test was used to compare the pro-Inflammatory gene expression levels between the subgroups. Data are mentioned in median with interquartile range. *p*-value, < 0.05. LBW – Low birth weight; NBW – Normal birth weight. **(B)** Comparison of expression of anti-inflammatory genes in subgroups. Mann Whitney U test was used to compare the anti-inflammatory gene expression levels between the subgroups. Data are mentioned in median with interquartile range. *p*-value, < 0.05. LBW – Low birth weight; NBW – Normal birth weight.

Supplementary Table 5 **(A)** Comparison of % DNA Methylation of Pro-Inflammatory Genes in Subgroups. Mann Whitney U test was used to compare the % DNA methylation level of pro-inflammatory gene between the subgroups. Data are mentioned in median with interquartile range. *p*-value, < 0.05. LBW – Low birth weight; NBW – Normal birth weight. **(B)** Comparison of % DNA methylation of anti-inflammatory genes in subgroups.

Supplementary Table 6 ROC Analysis of Inflammatory Cytokines for Neonatal Sepsis Discrimination. AUC, Area under the curve; CI, Confidence Interval 95%; *p*-value, <0.05.
