# Supplementary material for: Role of DNA methylation in regulating inflammatory cytokine expression in neonates with late-onset sepsis
Source: Front Immunol. 2026 Jan 26;16:1613333. doi: 10.3389/fimmu.2025.1613333 (PMC12883824; doi:10.3389/fimmu.2025.1613333)
Supplement: Supplementary Figure 1 — Representative result of MS-PCR analysis –inflammatory Cytokine Genes. The Supplementary Figures S1 shows the presence of methylation band in agarose gel electrophoresis. [file Presentation1.pptx]

## Slide 1
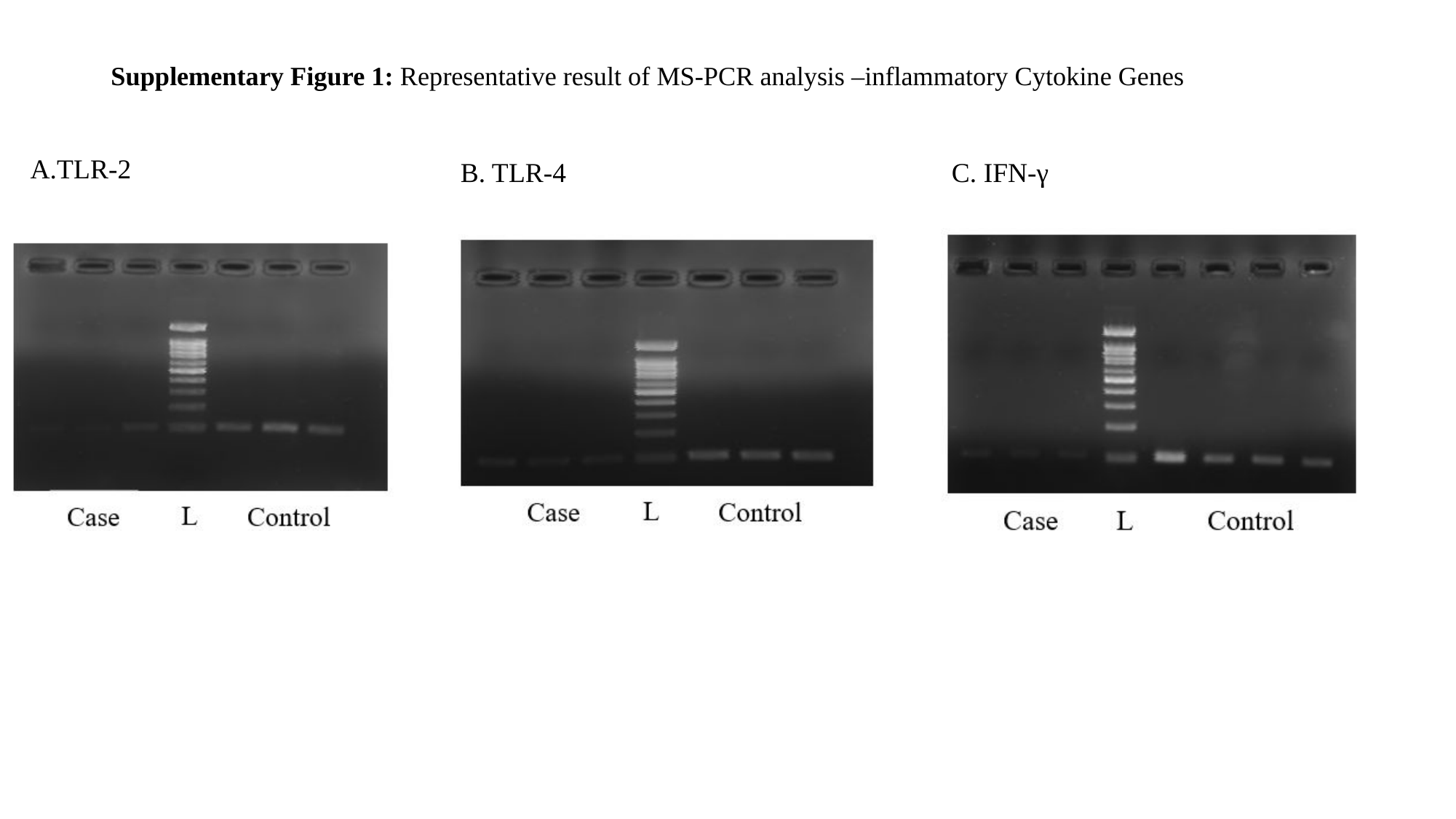

# Supplementary Figure 1: Representative result of MS-PCR analysis –inflammatory Cytokine Genes
A.TLR-2
B. TLR-4
C. IFN-γ

## Slide 2
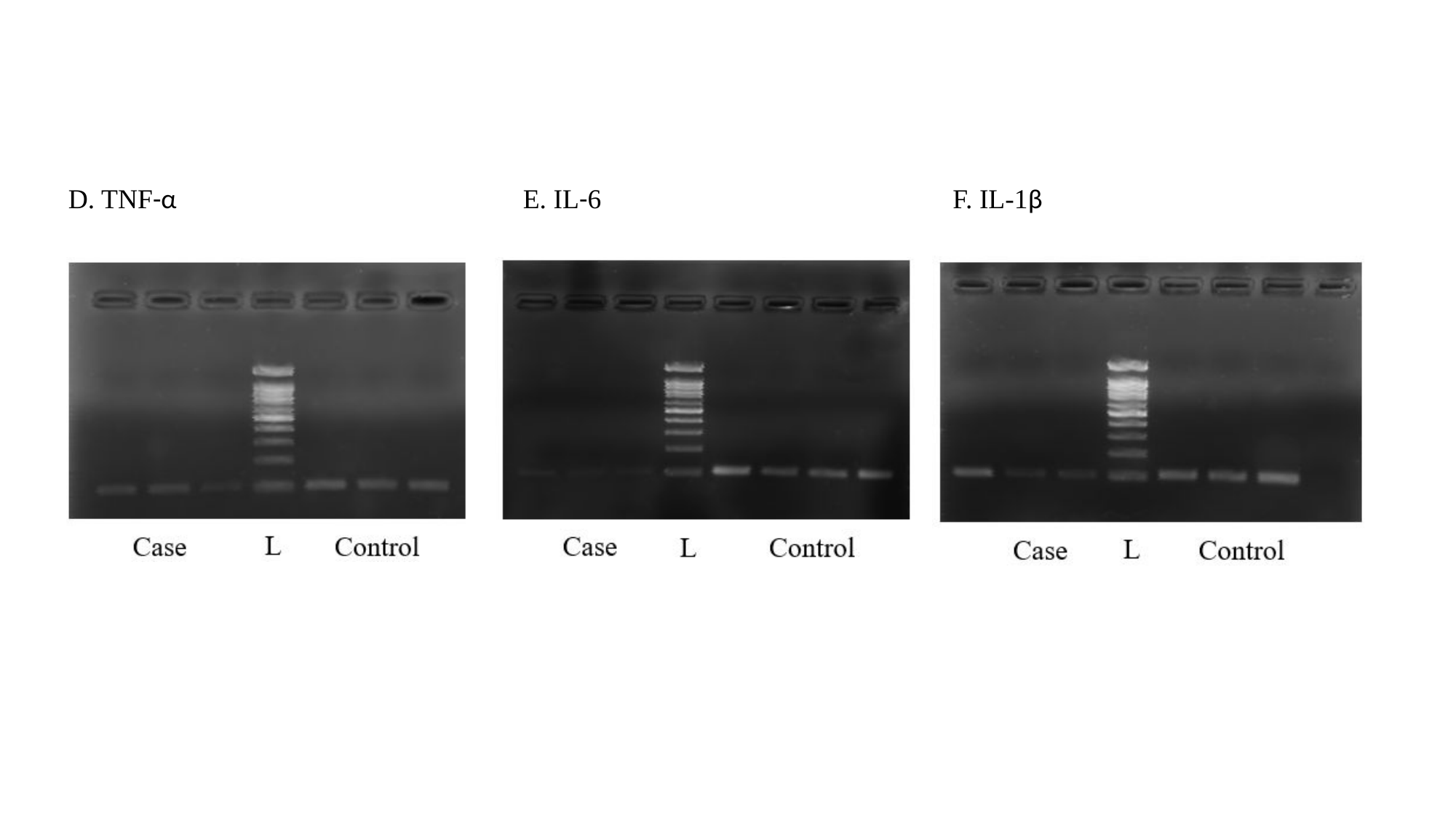

D. TNF-α
E. IL-6
F. IL-1β

## Slide 3
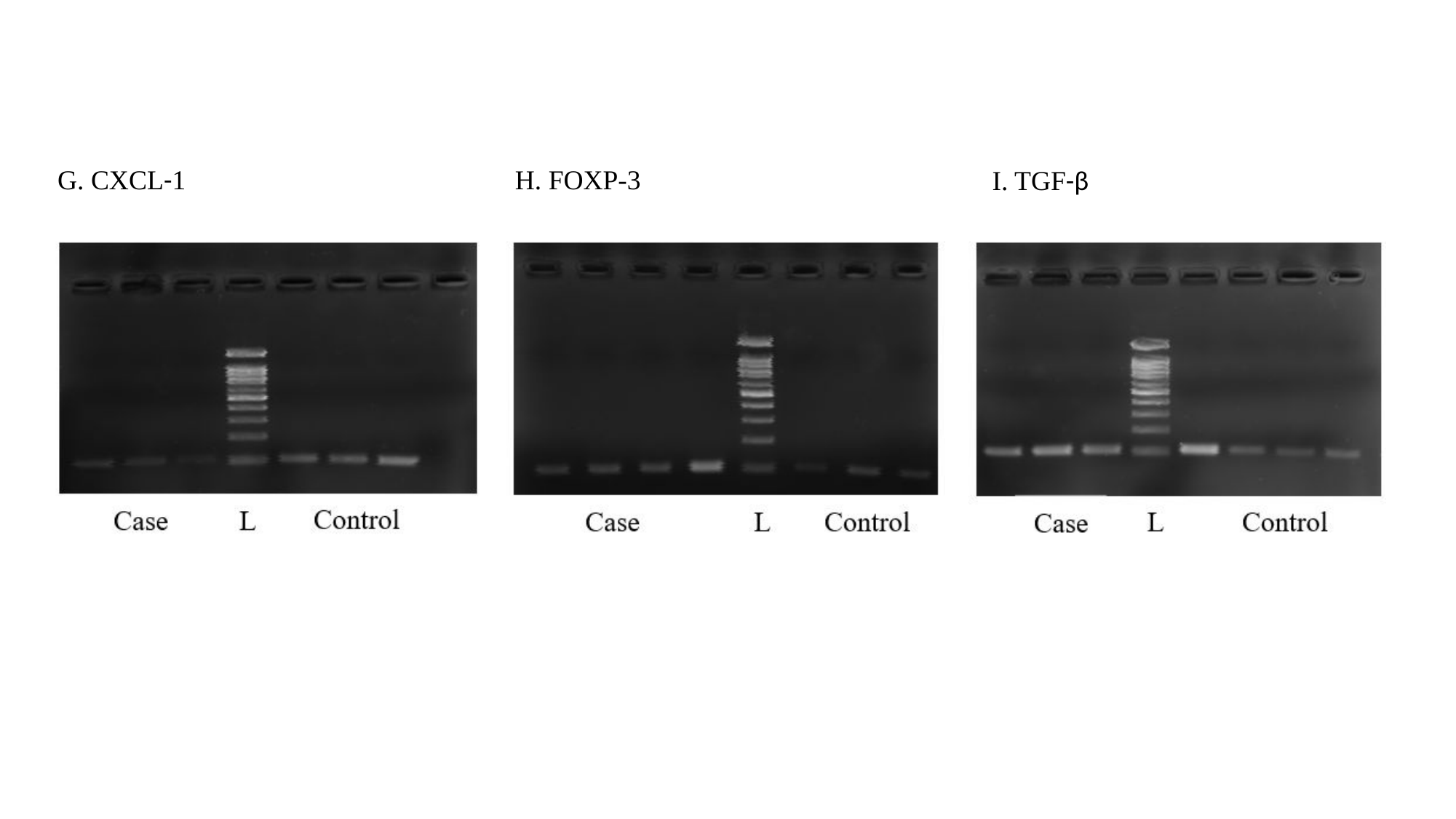

G. CXCL-1
H. FOXP-3
I. TGF-β

## Slide 4
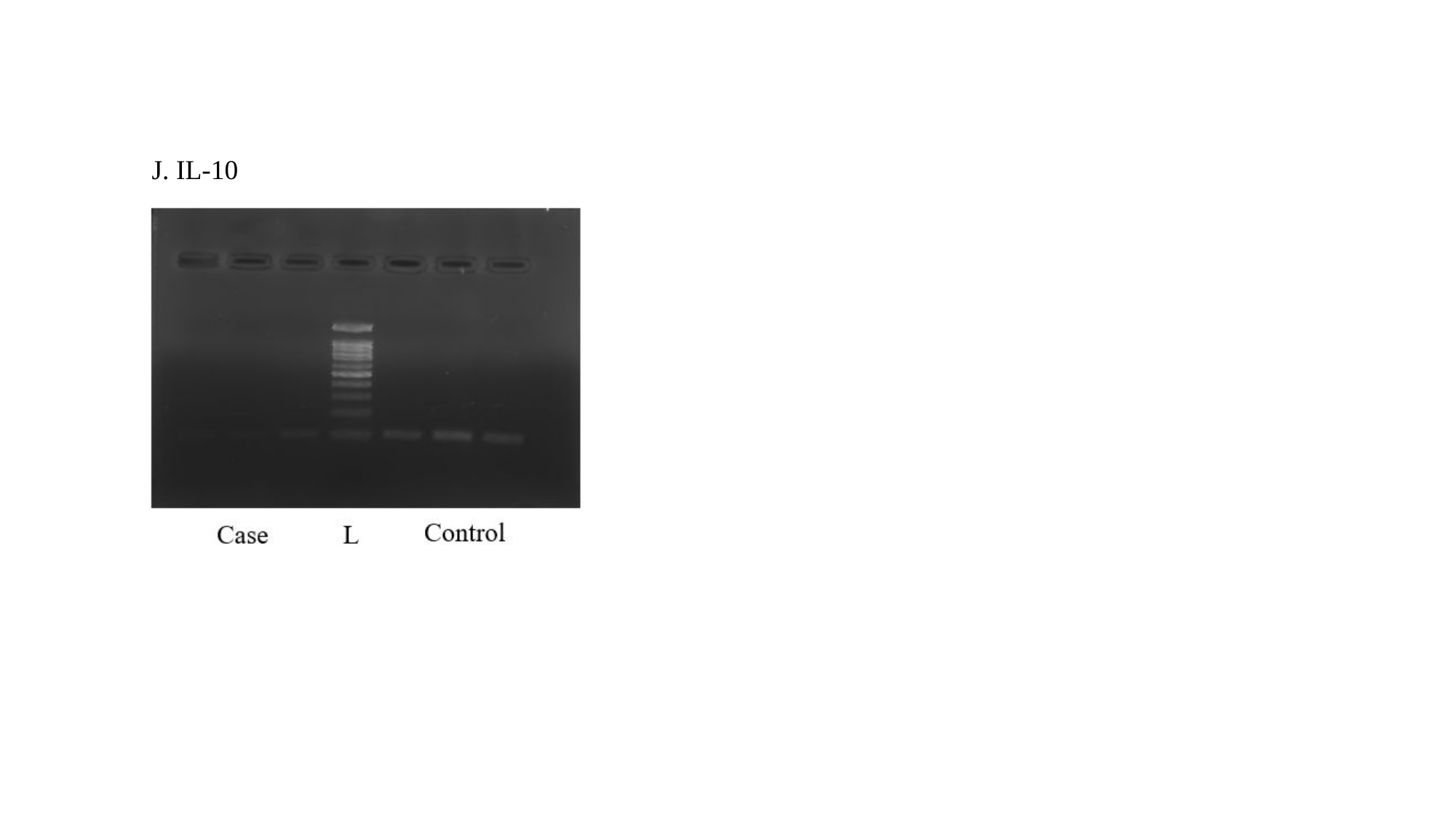

J. IL-10

## Slide 5
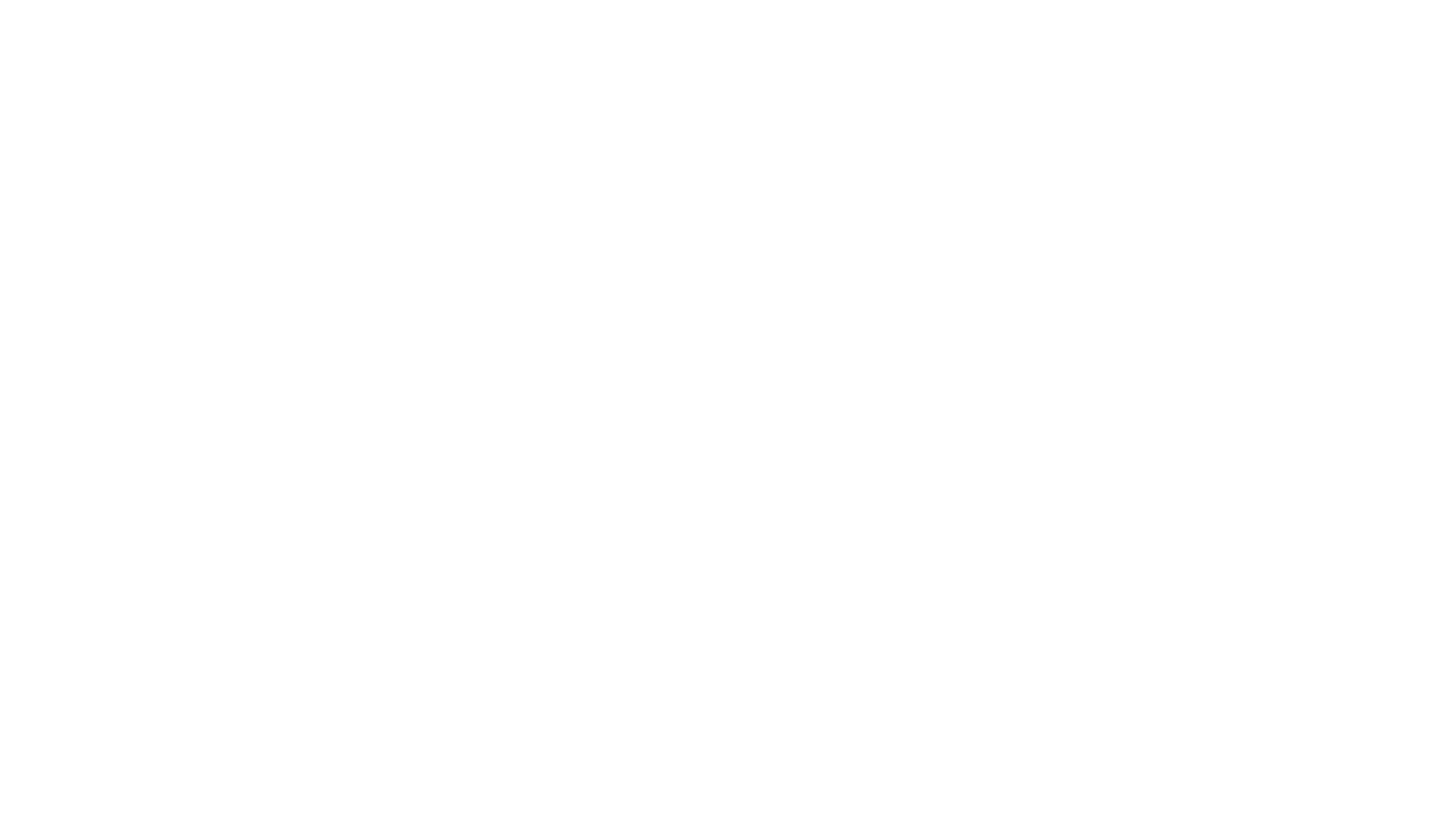

#

## Slide 6
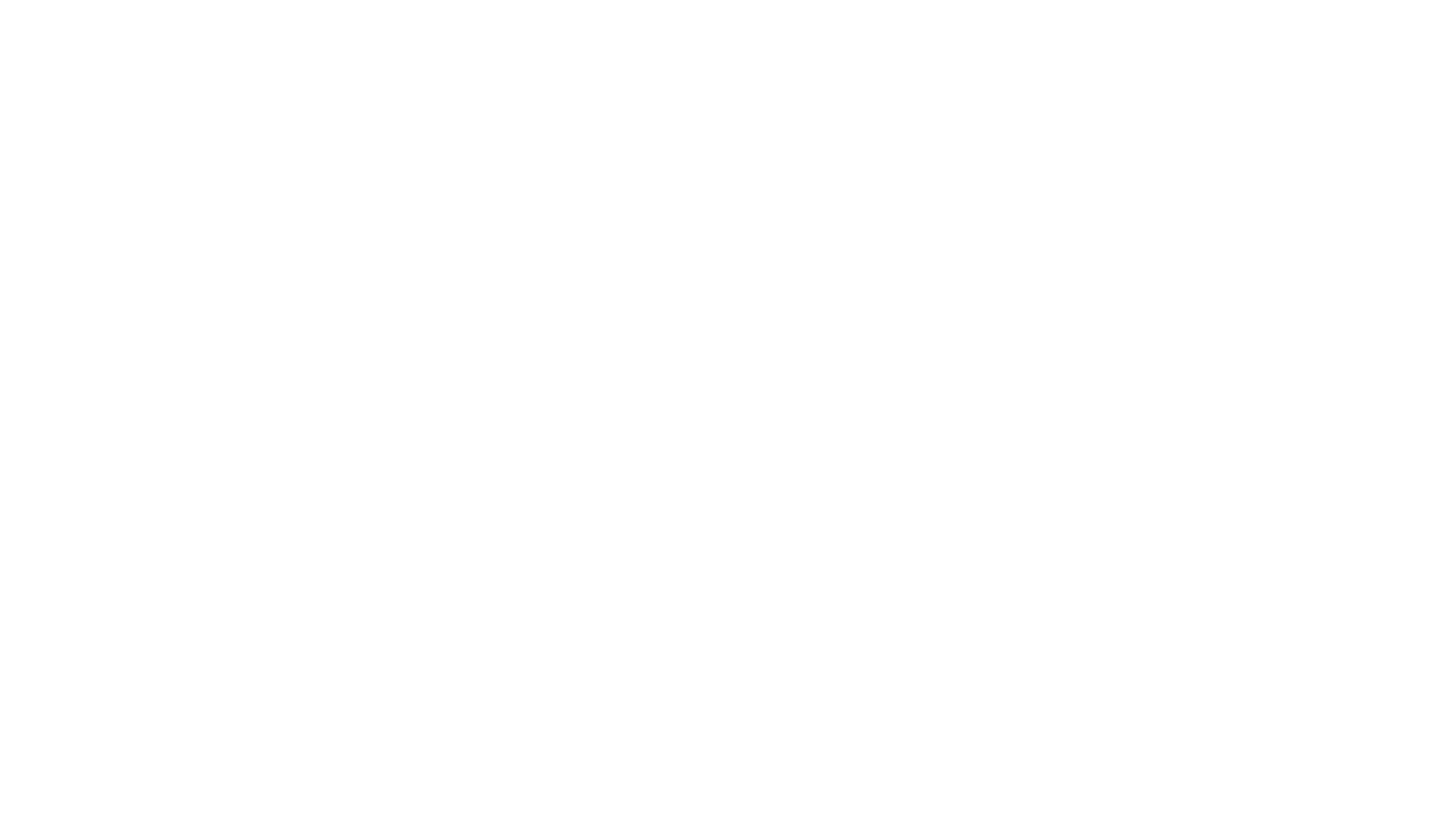

#

## Slide 7
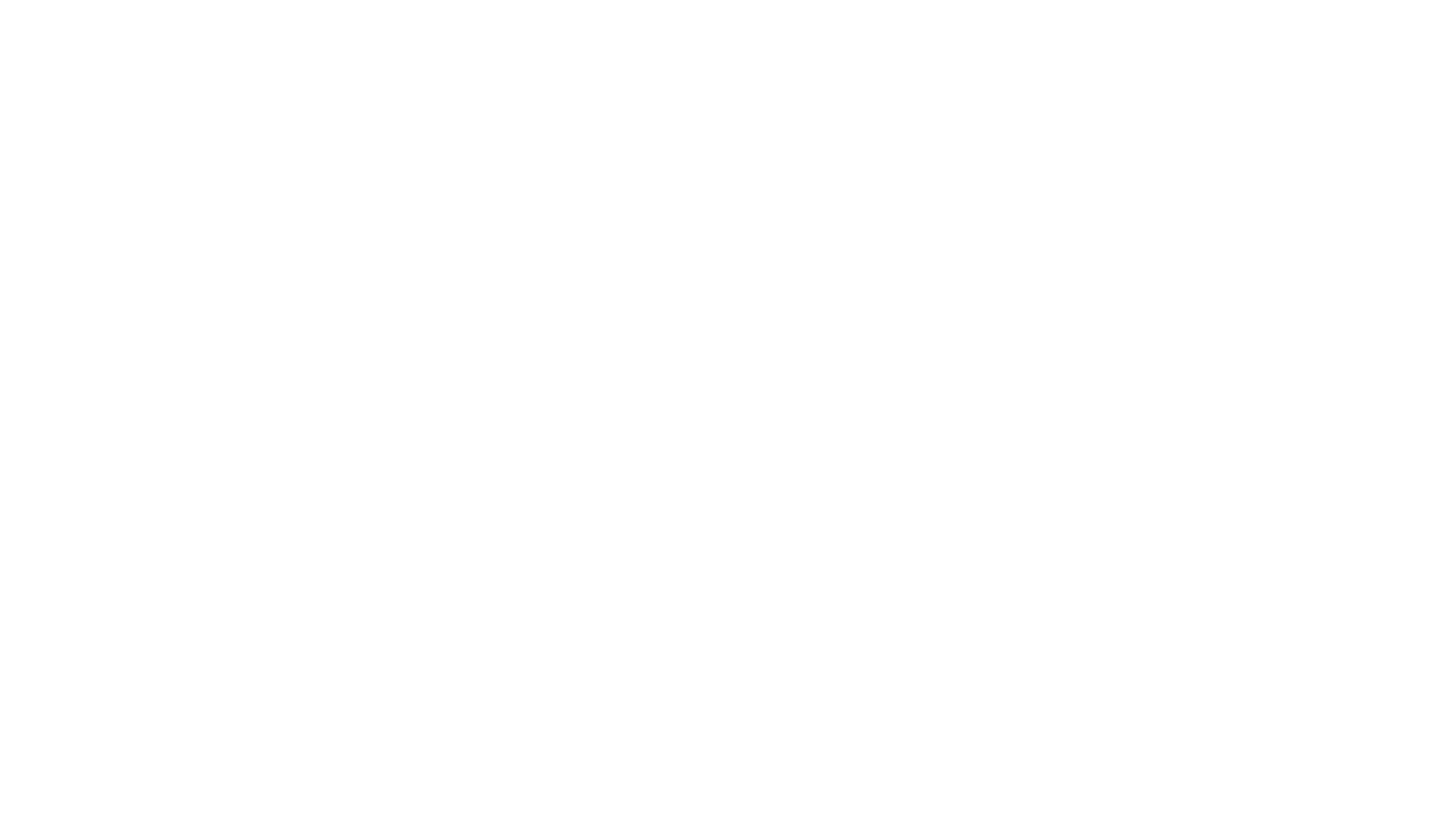

#

## Slide 8
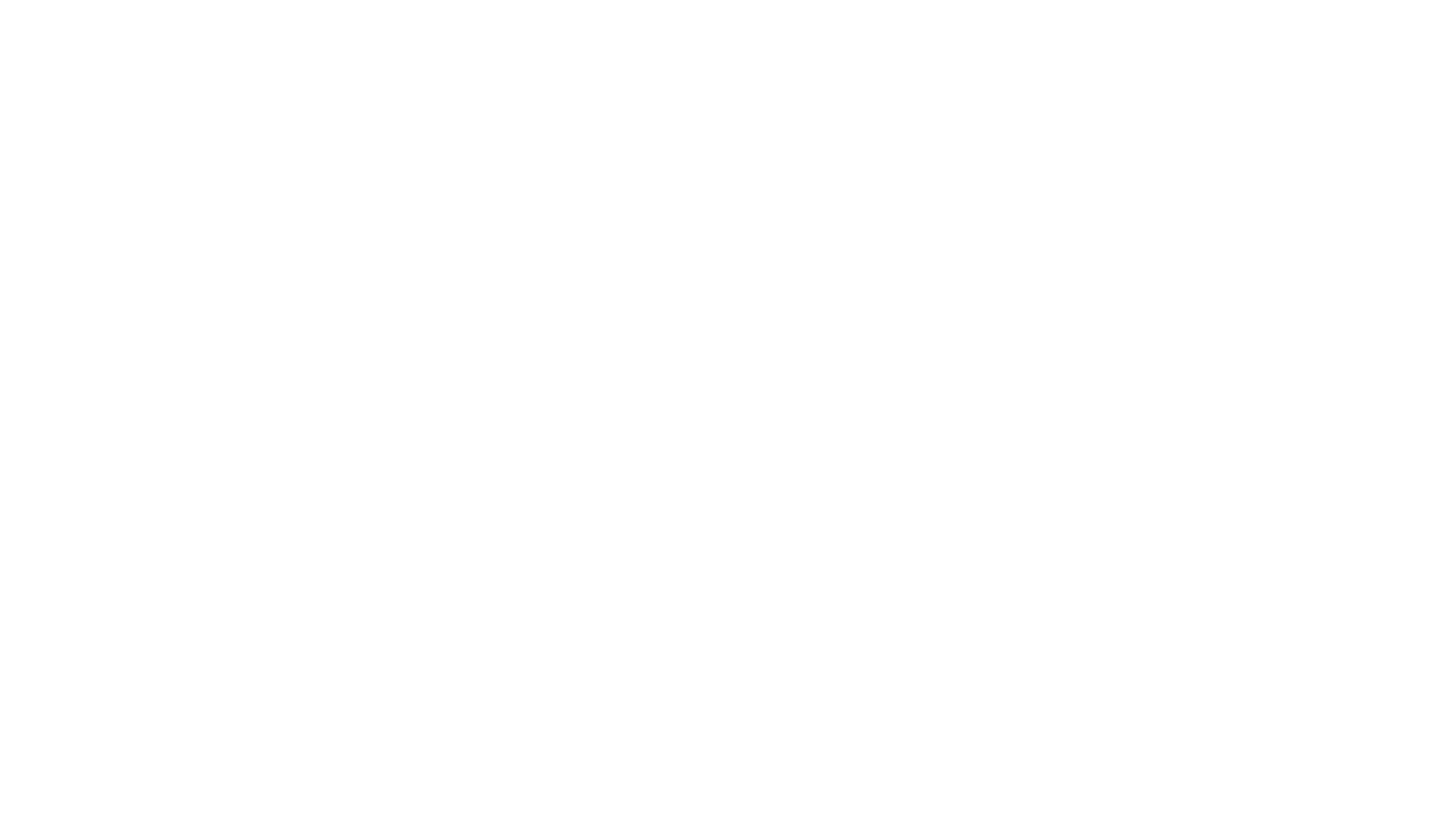

#

## Slide 9
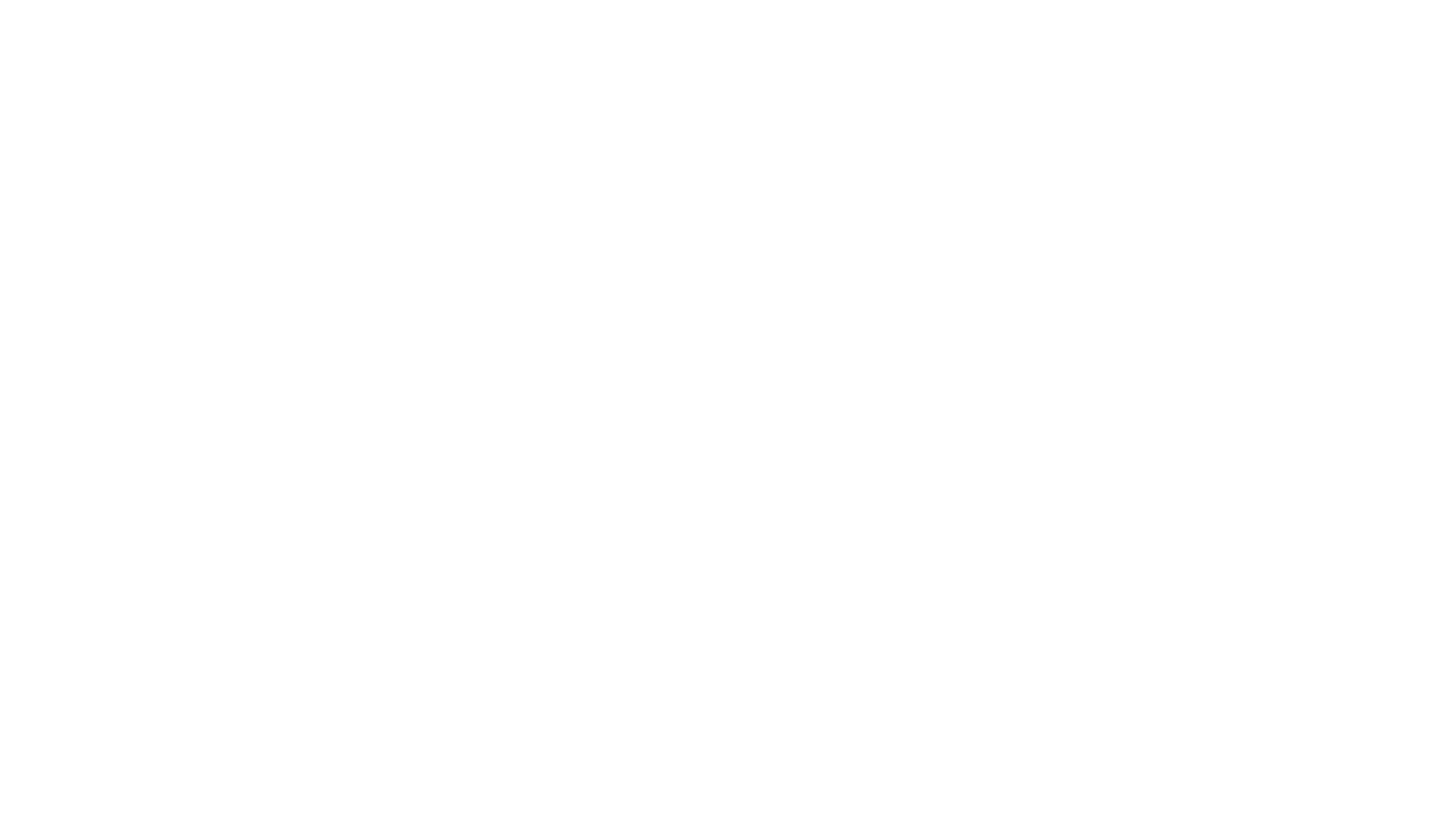

#

## Slide 10
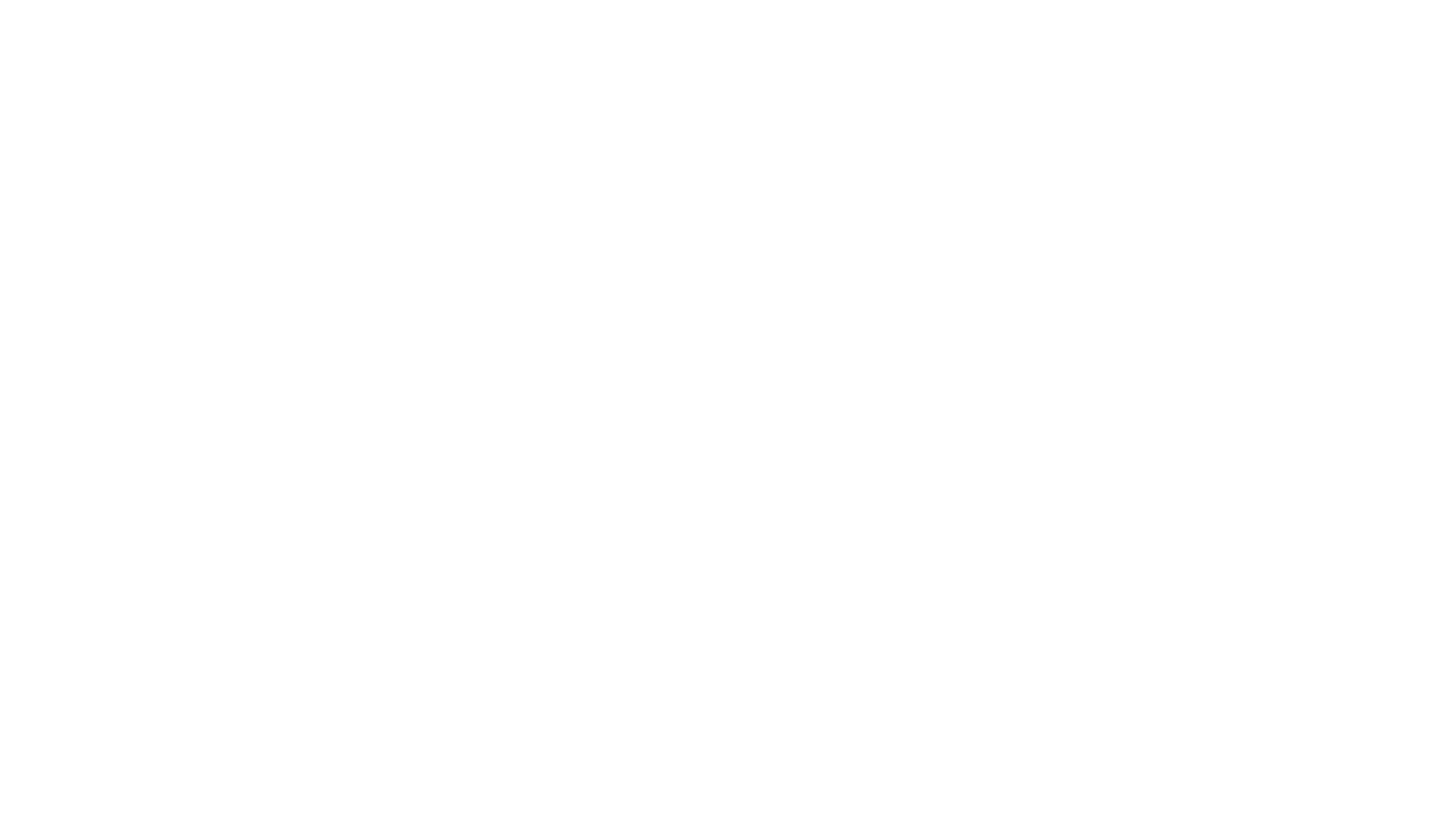

#
