# Supplementary material for: Role of DNA methylation in regulating inflammatory cytokine expression in neonates with late-onset sepsis
Source: Front Immunol. 2026 Jan 26;16:1613333. doi: 10.3389/fimmu.2025.1613333 (PMC12883824; doi:10.3389/fimmu.2025.1613333)
Supplement: Supplementary Table 1 — List of qRT-PCR Primers. The Supplementary Table S1 shows the primer details of pro- and anti-inflammatory genes and housekeeping genes. [file Table1.doc]

| **S. No** | **Gene Symbol** | **MSP Primers** | **Tm** | **Product Length** |
| --- | --- | --- | --- | --- |
| 1. | *TGFβMF* | TGTATATAGTTGTTGGTGGTATCGT | 52.1 | 165 |
|  | *TGFβMR* | TCCGAAATATAAATAATAATAACGTT |  |  |
|  | *TGFβUF* | TTGTATATAGTTGTTGGTGGTATTGT | 56.0 | 168 |
|  | *TGFβUR* | TCCAAAATATAAATAATAATAACATT |  |  |
| 2. | *FOXP3MF* | AGAGGTTTAAAAAGTGGGAGATTTC | 56.5 | 194 |
|  | *FOXP3MR* | ATTAACTCGCTACAACCATTATCGT |  |  |
|  | *FOXP3UF* | AGAGGTTTAAAAAGTGGGAGATTTT | 56.0 | 195 |
|  | *FOXP3UR* | TTAACTCACTACAACCATTATCATC |  |  |
| 3. | *IL10MF* | GTTTGAGAATGTTAGTTTTTAAACGA | 52.1 | 160 |
|  | *IL10MR* | CTAAACAAACCCTACCCTACCG |  |  |
|  | *IL10UF* | TTTGAGAATGTTAGTTTTTAAATGA | 52.1 | 162 |
|  | *IL10UR* | CTAAACAAACCCTACCCTACCAC |  |  |

**Supplementary Table 2. List MS-PCR Primers**

**Anti-inflammatory genes:**

**Pro-inflammatory genes:**

| **S. No** | **Gene Symbol** | **MSP Primers (5’ – 3’)** | **Tm** | **Product Length** |
| --- | --- | --- | --- | --- |
| 1. | *TLR2MF* | CGTGTAGTGTTAATGGGTTGC | 57.3 | 177 |
|  | *TLR2MR* | ACCAAAAAACGAAAAAAACGAC |  |  |
|  | *TLR2UF* | TGTGTAGTGTTAATGGGTTGTGG | 53.5 | 176 |
|  | *TLR2UR* | AAAAAACCAAAAAACAAAAAAAACA |  |  |
| 2. | *TLR4MF* | GTTTAGCGGTTTACATGATTTGAT | 56.1 | 173 |
|  | *TLR4MR* | CCCGCCCCTTCTTATAAAAAAACT |  |  |
|  | *TLR4UF* | GTTTAGTGGTTTATATGATTTGAT | 54.5 | 175 |
|  | *TLR4UR* | CCCACCCCTTCTTATAAAAAAACT |  |  |
| 3. | *IFNGMF* | AAGAGTTAATATTTTATTAGGGGGA | 52.1 | 237 |
|  | *IFNGMR* | TAAACTCCTTAAATCCTTTAAGGAT |  |  |
|  | *IFNGUF* | TGAAGAGTTAATATTTTATTAGGGTGA | 52.1 | 239 |
|  | *IFNGUR* | TAAACTCCTTAAATCCTTTAACAAT |  |  |
| 4. | *TNFprMF* | TAGAAGGTGTAGGGTTTATTATCGT | 52.1 | 141 |
|  | *TNFprMR* | TACCTTTATATATCCCTAAAACGAA |  |  |
|  | *TNFprUF* | TAGAAGGTGTAGGGTTTATTATTGT | 52.1 | 137 |
|  | *TNFprUR* | TACCTTTATATATCCCTAAAACAAA |  |  |
| 5. | *IL1BMF* | GTAGATTATAAGGTTAGGAGATCGA | 53.5 | 146 |
|  | *IL1BMR* | CCAAACTAAAATACAATAACACGAT |  |  |
|  | *IL1BUF* | GGTAGATTATAAGGTTAGGAGATTGA | 56.5 | 145 |
|  | *IL1BUR* | CCAAACTAAAATACAATAACACAAT |  |  |
| 6. | *IL6MF* | GGAGAAGATTTTAAAGATGTAGTCGT | 52.1 | 174 |
|  | *IL6MR* | AAAAACAAAAAAAACGCATACG |  |  |
|  | *IL6UF* | TTTTAGGAGAAGATTTTAAAGATGTAGTTG | 56.0 | 180 |
|  | *IL6UR* | CAAAAACAAAAAAAACACATACACA |  |  |
| 7. | *CXCL1MF* | GTTTTTTCGTTTTTTTTATAGTCGT | 52.5 | 203 |
|  | *CXCL1MR* | GCTAAATACCTACCCCAACCG |  |  |
|  | *CXCL1UF* | GTTTTTTTGTTTTTTTTATAGTTGT | 53.4 | 205 |
|  | *CXCL1UR* | ACACTAAATACCTACCCCAACCAC |  |  |

The supplementary table 2 shows the methylation primer details of pro- and anti-inflammatory genes.
