# Supplementary material for: Role of DNA methylation in regulating inflammatory cytokine expression in neonates with late-onset sepsis
Source: Front Immunol. 2026 Jan 26;16:1613333. doi: 10.3389/fimmu.2025.1613333 (PMC12883824; doi:10.3389/fimmu.2025.1613333)
Supplement: Supplementary Table 2 — List MS-PCR Primers. The Supplementary Table S2 shows the methylation primer details of pro- and anti-inflammatory genes and housekeeping genes. [file Table2.doc]

**Supplementary Table 1: List of qRT-PCR Primers**

**House-keeping Genes:**

| **S. No** | **Gene Symbol** | **qRT-PCR Primers (5’ – 3’)** | **Tm** |
| --- | --- | --- | --- |
| 1. | *HuPO* | GCAATGTTGCCAGTGTCTGT | 60 |
|  |  | GCCTTGACCTTTTCAGCAAG |  |
| 2. | *β2M* | ATGAGTATGCCTGCCGTGTG | 60 |
|  |  | CCAAATGCGGCATCTTCAAAC |  |

**Anti-inflammatory genes:**

| **S. No** | **Gene Symbol** | **qRT-PCR Primers (5’ – 3’)** | **Tm** |
| --- | --- | --- | --- |
| 1. | *TGFβ* | TCCAGGCTCCAAATGTAGG | 60 |
|  |  | GGACACCAACTATTGCTTCAG |  |
| 2. | *IL-10* | TCATCTCAGAACAAGGCTTGGC | 60 |
|  |  | CGAGATGCCTTCAGCAGAGTG |  |
| 3. | *FOXP3* | CAGCACATTCCCAGAGTTCCTC | 60 |
|  |  | GCGTGTGAACCAGTGGTAGATC |  |

**Pro-inflammatory gene**s:

| **S. No** | **Gene Symbol** | **qRT-PCR Primers (5’ – 3’)** | **Tm** |
| --- | --- | --- | --- |
| 1. | *TLR2* | GGTCTTGGTGTTCATTATCTTC | 60 |
|  |  | TCTCCCATTTCCGTCTTTTT |  |
| 2. | *TLR4* | GGTCAGACGGTGATAGCGAG | 56 |
|  |  | ATTAGGAACCACCTCCACGC |  |
| 3. | *IFN-γ* | ATTCGGTAACTGACTTGAATGTCC | 60 |
|  |  | CTCTTCGACCTCGAAACAGC |  |
| 4. | *TNF-α* | TGGCCCAGGCAGTCAGA | 60 |
|  |  | GGTTTGCTACAACATGGGCTACA |  |
| 5. | *IL-1β* | GCCCTAAACAGATGAAGTGCTC | 60 |
|  |  | GAACCAGCATCTTCCTCAG |  |
| 6. | *IL6* | TGCAATAACCACCCCTGACC | 62 |
|  |  | GTGCCCATGCTACATTTGCC |  |
| 7. | *CXCL1* | GCCAGTGCTTGCAGACCCT | 60 |
|  |  | GGCTATGACTTCGGTTTGGG |  |

The supplementary table 1 shows the primer details of pro- and anti-inflammatory genes and house keeping genes.
