# Supplementary material for: Role of DNA methylation in regulating inflammatory cytokine expression in neonates with late-onset sepsis
Source: Front Immunol. 2026 Jan 26;16:1613333. doi: 10.3389/fimmu.2025.1613333 (PMC12883824; doi:10.3389/fimmu.2025.1613333)
Supplement: Supplementary Table 3 — List Bisulfite Pyrosequencing Primers. The Supplementary Table S2 shows the bisulfite pyrosequencing primer details of pro- and anti-inflammatory genes and housekeeping genes. [file Table3.doc]

**Supplementary Table 6.** ROC Analysis of Inflammatory Cytokines for Neonatal Sepsis Discrimination

| **Cytokine** | **AUC** | **95% CI** | **Cut-off** | **Sensitivity (%)** | **Specificity (%)** | ***p-*value** |
| --- | --- | --- | --- | --- | --- | --- |
| IFN-γ | 0.993 | 0.977-1.000 | 49.3 | 100 | 98 | **<0.0001** |
| TNF-α | 0.953 | 0.913-0.992 | 124.8 | 90 | 83 | **<0.0001** |
| IL-10 | 0.997 | 0.990-1.000 | 24.3 | 97 | 100 | **<0.0001** |
| TGF-β | 0.853 | 0.761-0.944 | 43 | 80 | 83 | **<0.0001** |

AUC, Area under the curve; CI, Confidence Interval 95%; *p*-value, <0.05.
