# Supplementary material for: Role of DNA methylation in regulating inflammatory cytokine expression in neonates with late-onset sepsis
Source: Front Immunol. 2026 Jan 26;16:1613333. doi: 10.3389/fimmu.2025.1613333 (PMC12883824; doi:10.3389/fimmu.2025.1613333)
Supplement: Supplementary Table 4 — (A) Comparison of Pro-Inflammatory Gene Expression in Subgroups. Mann Whitney U test was used to compare the pro-Inflammatory gene expression levels between the subgroups. Data are mentioned in median with interquartile range. p-value, < 0.05. LBW – Low birth weight; NBW – Normal birth weight. (B) Comparison of expression of anti-inflammatory genes in subgroups. Mann Whitney U test was used to compare the anti-inflammatory gene expression levels between the subgroups. Data are mentioned in median with interquartile range. p-value, < 0.05. LBW – Low birth weight; NBW – Normal birth weight. [file Table4.doc]

**Supplementary Table 3A.** Comparison of % DNA Methylation of Pro-Inflammatory Genes in Subgroups

| **Group** | **No** | **% DNA Methylation (median, IQR)** | | | | | | | |
| --- | --- | --- | --- | --- | --- | --- | --- | --- | --- |
|  |  | ***TLR2*** | ***TLR4*** | ***TNF-α*** | ***IFN-γ*** | ***IL-1β*** | ***IL-6*** | ***CXCL1*** |  |
| Pre-term | 24 | 10 (10-25) | 10 (10-25) | 17.5 (10-25) | 10 (10-25) | 10 (10-25) | 10 (10-25) | 10 (10-25) |  |
| Term | 16 | 25 (10-43.75) | 10 (10-25) | 10 (10-25) | 10 (10-25) | 10 (10-21.25) | 10 (10-25) | 10 (10-25) |  |
| ***p* value** | | 0.04 | 0.9 | 0.8 | 0.9 | 0.5 | 0.2 | 0.6 |  |
| LBW | 24 | 10 (10-25) | 10 (10-25) | 25 (10-25) | 10 (10-25) | 10 (10-25) | 10 (10-25) | 10 (10-25) |  |
| NBW | 16 | 25 (10-25) | 10 (10-25) | 10 (10-25) | 10 (10-25) | 10 (10-10) | 10 (10-25) | 10 (10-25) |  |
| ***p* value** | | 0.2 | 0.5 | 0.4 | 0.9 | 0.1 | 0.5 | 0.2 |  |
| Survivors | 22 | 10 (10-25) | 10 (10-13.75) | 17.5 (10-25) | 10 (10-25) | 10 (10-25) | 10 (10-25) | 10 (10-25) |  |
| Non-survivors | 18 | 10 (10-25) | 10 (10-25) | 10 (10-25) | 10 (10-25) | 10 (10-25) | 25 (25-25) | 10 (10-25) |  |
| ***p* value** | | 0.3 | 0.1 | 0.8 | 0.6 | 0.9 | **0.04** | 0.3 |  |

Mann Whitney U test was used to compare the % DNA methylation level of pro-inflammatory gene between the subgroups. Data are mentioned in median with interquartile range. *p*-value*,* <0.05. LBW – Low birth weight; NBW – Normal birth weight.

**Supplementary Table 3B.** Comparison of % DNA methylation of anti-inflammatory genes in subgroups

| **Group** | **No** | **% DNA Methylation, median (IQR)** | | |
| --- | --- | --- | --- | --- |
|  |  | ***IL-10*** | ***TGF-β*** | ***FOXP3*** |
| Pre-term | 24 | 17.5 (10-25) | 75 (50-75) | 75 (50-75) |
| Term | 16 | 25 (10-25) | 50 (50-75) | 75 (50-75) |
| ***p* value** | | 0.3 | **0.02** | 0.7 |
| LBW | 24 | 25 (10-25) | 62.5 (50-75) | 75 (50-75) |
| NBW | 16 | 25 (10-25) | 75 (50-75) | 75 (50-75) |
| ***p* value** | | 0.9 | 0.5 | 0.7 |
| Survivors | 22 | 25 (10-25) | 75 (50-75) | 75 (68.75-75) |
| Non-survivors | 18 | 25 (10-25) | 50 (50-75) | 50 (50-75) |
| ***p* value** | | 0.9 | 0.3 | 0.06 |

Mann Whitney U test was used to compare the % DNA methylation level of anti-inflammatory gene between the subgroups. Data are mentioned in median with interquartile range. *p*-value*,* <0.05. LBW – Low birth weight; NBW – Normal birth weight.
