# Supplementary material for: Role of DNA methylation in regulating inflammatory cytokine expression in neonates with late-onset sepsis
Source: Front Immunol. 2026 Jan 26;16:1613333. doi: 10.3389/fimmu.2025.1613333 (PMC12883824; doi:10.3389/fimmu.2025.1613333)
Supplement: Supplementary Table 5 — (A) Comparison of % DNA Methylation of Pro-Inflammatory Genes in Subgroups. Mann Whitney U test was used to compare the % DNA methylation level of pro-inflammatory gene between the subgroups. Data are mentioned in median with interquartile range. p-value, < 0.05. LBW – Low birth weight; NBW – Normal birth weight. (B) Comparison of % DNA methylation of anti-inflammatory genes in subgroups. [file Table5.doc]

**Supplementary Table 4A.** Comparison of Pro-Inflammatory Gene Expression in Subgroups

| **Group** | **No** | **Gene expression, median (IQR)** | | | | | | | |
| --- | --- | --- | --- | --- | --- | --- | --- | --- | --- |
|  |  | ***TLR2*** | ***TLR4*** | ***NFκB*** | ***TNF-α*** | ***IFN-γ*** | ***IL-1β*** | ***IL-6*** | ***CXCL1*** |
| Pre-term | 26 | 4.3 (3.5-5.1) | 6.1 (4.1-11.3) | 5.3 (3.7-7.8) | 6.0 (4.5-7.8) | 5.9 (4.6-8.8) | 5.7 (4.2-7.8) | 8.5 (5.7-10.7) | 5.2 (3.6-6.6) |
| Term | 16 | 5.1 (3.5- 6.4) | 7.6 (4.9-8.5) | 4.8 (4.1-5.8) | 5.2 (4.1-7.1) | 7.5 (5.4-9.8) | 5.9 (4.2-7.9) | 7.8 (5.3-13.3) | 6.2 (4.2-7.1) |
| ***p* value** | | 0.1 | 0.4 | 0.4 | 0.2 | 0.3 | 0.9 | 0.5 | 0.2 |
| LBW | 24 | 4.3 (3.2-5.4) | 5.5 (4.1-9.9) | 5.1 (3.7-7.2) | 5.4 (4.5-7.8) | 6.3 (4.9-9.5) | 5.0 (4.1-8.1) | 7.6 (5.7-10.9) | 5.3 (4.3-6.7) |
| NBW | 18 | 4.7 (3.9-5.7) | 7.7 (5.0-11.4) | 5.2 (4.3-7.3) | 5.5 (4.2-7.1) | 7.2 (4.7-9.3) | 6.5 (4.4-7.5) | 9.8 (5.5-13.3) | 5.7 (3.3-7.1) |
| ***p* value** | | 0.2 | 0.2 | 0.5 | 0.6 | 0.9 | 0.8 | 0.2 | 0.7 |
| Survivors | 25 | 4.3 (3.1-5.6) | 5.5 (3.8-9.0) | 5.8 (3.6-7.6) | 5.2 (4.1-7.3) | 6.1 (4.8-8.3) | 5.7 (4.1-8.1) | 6.5 (4.9-11.3) | 5.3 (3.6-6.9) |
| Non-survivors | 17 | 4.5 (4.1-5.7) | 7.2 (5.1-11.8) | 4.7 (3.9-5.3) | 6.0 (4.7-7.8) | 7.9 (5.3-10.1) | 5.8 (4.2-7.7) | 10.8 (7.6-12.3) | 5.4 (4.1-6.8) |
| ***p* value** | | 0.3 | 0.1 | 0.3 | 0.3 | **0.05** | 0.8 | **0.01** | 0.8 |

Mann Whitney U test was used to compare the pro-Inflammatory gene expression levels between the subgroups. Data are mentioned in median with interquartile range. *p*-value*,* <0.05. LBW – Low birth weight; NBW – Normal birth weight.

**Supplementary Table 4B**. Comparison of expression of anti-inflammatory genes in subgroups

| **Group** | **No** | **Gene expression, median (IQR)** | | |
| --- | --- | --- | --- | --- |
|  |  | ***IL-10*** | ***TGF-β*** | ***FOXP3*** |
| Pre-term | 26 | 3.2 (2.4-5.7) | 1.7 (1.3-2.3) | 1.8 (1.3-2.8) |
| Term | 16 | 4.5 (3.1-5.4) | 2.4 (1.7-3.6) | 1.6 (0.9-2.6) |
| ***p* value** | | 0.2 | **0.03** | 0.4 |
| LBW | 24 | 3.3 (2.4-6.0) | 1.7 (1.3-2.5) | 1.9 (1.3-3.3) |
| NBW | 18 | 4.1 (3.0-5.3) | 2.0 (1.7-2.8) | 1.5 (0.9-2.3) |
| ***p* value** | | 0.6 | 0.3 | 0.09 |
| Survivors | 25 | 4.0 (2.6-5.7) | 1.9 (1.4-2.8) | 1.6 (1.1-3.4) |
| Non-survivors | 17 | 3.6 (2.7-4.9) | 1.9 (1.2-2.7) | 2.0 (1.3-2.8) |
| ***p* value** | | 0.4 | 0.4 | 0.6 |

Mann Whitney U test was used to compare the anti-inflammatory gene expression levels between the subgroups. Data are mentioned in median with interquartile range. *p*-value*,* <0.05. LBW – Low birth weight; NBW – Normal birth weight.
